# Thrombotic microangiopathy mediates poor prognosis among lupus nephritis *via* complement lectin and alternative pathway activation

**DOI:** 10.3389/fimmu.2022.1081942

**Published:** 2022-12-13

**Authors:** Binshan Zhang, Guolan Xing

**Affiliations:** Department of nephrology, First Affiliated Hospital of Zhengzhou University, Zhengzhou, China

**Keywords:** lupus nephritis, thrombotic microangiopathy, lectin pathway, alternative pathway, complement

## Abstract

**Objective:**

The pathogenesis of thrombotic microangiopathy (TMA) in lupus nephritis (LN) remains complicated. This study aimed to detect the deposition of complement lectin pathway (LP) and alternative pathway (AP) components in renal tissues, then evaluate the clinicopathological characteristics and risk factors for renal survival between patients with or without TMA in LN cohorts.

**Methods:**

We included 79 patients with biopsy-proven LN-associated TMA and matched the same number of LN patients without TMA as the control group. The deposition of mannose binding lectin (MBL), MBL-associated serine proteases 1/3 (MASP1/3), complement factor B (CFB), complement factor D (CFD), C4d, and von Willebrand factor (VWF) in renal tissue was assessed by immunohistochemistry and immunofluorescence. Besides, co-localization of C5b-9 and CD34 was detected by confocal microscopy.

**Results:**

In our retrospective cohort, the incidence of acute kidney injury (30% vs. 14%, p = 0.013), acute hemodialysis (35% vs. 5%, p < 0.001), and interstitial fibrosis (43% vs. 13%, p < 0.001) is higher in the TMA, compared with the control group. Despite aggressive steroids pulse, plasma exchange, and immunosuppressive therapy among TMA group, they still had significantly inferior 3-year renal survival rates (68% vs. 89%, p = 0.002) than those in the non-TMA group. COX regression analysis identified that TMA (HR 4.807, 95% CI [2.052, 11.263], p < 0.001) is a risk factor in LN. MBL, MASP1/3, CFB, CFD, C4d, and VWF deposited along the glomerulus among LN, while TMA had stronger staining intensity and deposition. The co-localized expression of CD34 and C5b-9 in the endothelial cells was also observed in the renal tissues.

**Conclusions:**

TMA is an independent risk factor for renal survival in LN patients. Moreover, LP and AP activation are involved in the pathogenesis of LN-associated TMA.

## Introduction

Systemic lupus erythematosus (SLE) is an autoimmune disease characterized by the overproduction of autoantibodies, which has various clinical manifestations and affects multiple tissues and organs, of which the renal involvement is the most important predictor of morbidity and mortality (1, 2). The histological damage in lupus nephritis (LN) is often associated with different treatment responses and outcomes (3). In addition to common glomerulonephritis, renal vasculopathies closely related to prognosis, including vascular immune complex deposits, arterial sclerosis, noninflammatory necrotizing vasculopathy, thrombotic microangiopathy (TMA), and true renal vasculitis, have attracted great attention (4–6). TMA-associated with LN has a high probability of developing end-stage renal disease (ESRD) and death (7, 8).

The etiology of LN is complicated and include extrarenal and intrarenal factors, such as inheritance, sex hormone, virus, immunity, and environment (9). Hypocomplementemia is often thought to be related to the activity of renal disease. However, the effects of deficiency and over activation of the complement system in SLE and TMA have not been fully elucidated. Complement activation can be initiated through 3 different pathways. Immune complexes deposited in the glomerulus and peritubular capillaries activate the complement classical pathway (CP), which is thought to be the dominant pathway in LN (9). Interestingly, C1q deficiency is strongly relevant to development of SLE (10). The lectin pathway (LP) is activated when mannose binding lectin (MBL), as one of the pattern recognition molecules, binds to bacterial carbohydrate motifs and engage the MBL-associated serine proteases (MASPs). MASPs are indispensable for LP and alternative pathway (AP) activation. MASP-1 autoactivates firstly, then it activates MASP-2 (11). MASP-2 subsequently cleave C2 and C4 to form the C3 convertase (C4b2a) (12). Furthermore, MASP-3 can exclusively activate pro-factor D in normal resting human blood to regulate AP activity (13, 14). Complement factor D (CFD) cleaves factor B to generate the AP convertase (C3bBb) (12). Three complement pathways eventually form the C5b-9 membrane attack complex to promote cells lysis (15).

Growing evidence suggests that LP and AP are involved in the pathogenesis of LN and TMA (16). It has been reported that most LN patients presented MBL deposition and had higher urinary protein than those negative (17). MBL2 gene polymorphism also influences vulnerability to SLE (18). Recently, J Laurence et al. discovered that elevated MASP-2 levels in TMA patients are associated with microvascular endothelial cell (MVEC) damage and can be restrained by narsoplimab (anti-MASP-2 antibody) *in vitro* (19). CFD deficiency in MRL/lpr mice has a protective effect on renal disease due to the lack of AP activation (20). Additionally, AP overactivation leading to MVEC damage and microvascular thrombosis is a common mechanism that induces TMA (21).

The complement component deposition of LP and AP in TMA-associated with LN patients has not been studied before. Therefore, this research detected the expression of relevant indicators in renal tissues to explore the pathogenesis of LN with TMA. In addition, we analyzed the clinicopathological characteristics and risk factors affecting prognosis between patients with or without TMA in LN cohorts.

## Materials and methods

### Patients selection

A total of 2130 Chinese underwent renal biopsy at The First Affiliated Hospital of Zhengzhou University between January 2012 and July 2021 were enrolled. Of the 2130 individuals, patients who fulfilled the following criteria were defined as LN-TMA: (1) meet the 2019 European League Against Rheumatism/American College of Rheumatologic Classification Criteria (EULAR/ACR) for the diagnosis of SLE (22). (2) LN was diagnosed according to The 2018 International Society of Nephrology/Renal Pathology Society (ISN/PRS) pathological classification (23). (3) evaluation of TMA pathological features under light microscopy (LM) and electron microscopy (EM). The exclusion criteria were as follows: (1) The total number of glomeruli was less than 10 under LM. (2) inadequate clinical information data (no information on proteinuria, serum creatinine, etc.). (3) insufficient time or loss to follow-up. Ultimately, 79 patients met the criteria of this research. According to gender, age, and LN classes, we matched the same number of LN patients without TMA as the control group. The retrospective study protocol was approved by the ethics committees of The First Affiliated Hospital of Zhengzhou University.

### Clinical and laboratory data

The collection of complete clinical information of enrolled patients was performed at the time of renal biopsy and listed below: (1) General information: age, gender, duration of SLE/LN, systolic and diastolic blood pressure (SBP and DBP), leukocytes, hemoglobin (Hb), platelets (PLT), 24-h urinary protein, hematuria, serum albumin (Alb), serum creatinine (SCr), total cholesterol and triglyceride; (2) LN-related specific indicators: serum C3 and C4 levels, C-reactive protein, erythrocyte sedimentation rate (ESR), antinuclear antibody(ANA), anti-dsDNA antibody (A-dsDNA), fever, rash, mucosal ulcers, arthritis, serositis, neurological symptoms, the SLE Disease Activity Index (SLEDAI) (24), acute kidney injury(AKI), acute hemodialysis and acute heart failure (AHF). The above serological examinations which were measured in a fasting state and performed in the central laboratory in our hospital are routinely tested in clinical practice. During the follow-up period, the interval between patients visit is 3-6 months. The above parameters were collected during each visit.

### Renal histopathology

The paraffin-embedded kidney tissues were stained with hematoxylin-eosin (HE), periodic acid-schiff (PAS), masson, and periodic acid-silver metheramine (PASM), respectively. Three-micrometer cryostat sections were stained with anti-human IgG, IgM, IgA, C3, C4, and C1q for immunofluorescence (IF). A routine read of every renal biopsy specimen was performed by two professional pathologists through LM, IF, and EM, and reported according to the 2018 ISN/PRS classification (23).

**Figure 1:**
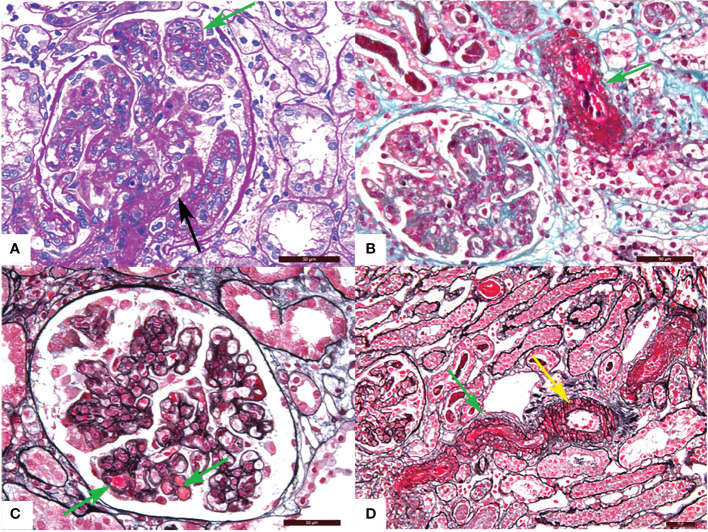
Representative light microscopy images of renal thrombotic microangiopathy (TMA). **(A)** Proliferation of glomerular mesangial cell and endothelial cell (green arrow), thickening of glomerular basement membrane and formation of the double track sign (black arrow) (PAS). **(B)** Fibrinous necrosis of the arteriole (green arrow) (Masson). **(C)** Microthrombosis in the glomerular capillary (green arrow) (PASM-Masson). **(D)** Arteriolar thrombosis (green arrow) and intimal myxoid oedema (yellow arrow) (PASM-Masson). PAS, periodic acid-schiff. PASM, periodic acid-silver methenamine. Scale bars represent 50μm.

**Figure 2:**
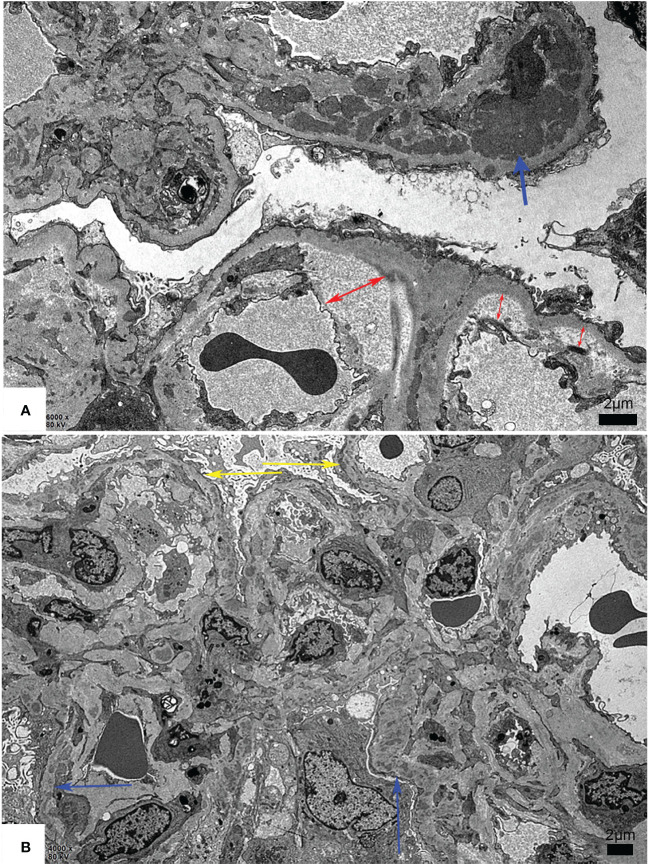
Representative electron microscopy changes of renal thrombotic microangiopathy. **(A)** The dense deposition (blue arrow) and mild, moderate widening of the loose layer (red arrow) in the basement membrane. **(B)** The formation of the double track sign (yellow arrow) and dense deposition in the basement membrane (blue arrow). Scale bars represent 2μm.

TMA is divided into acute and chronic lesions, which involve the glomeruli, renal tubules, and interstitial blood vessels. Under the LM, glomerular manifests as capillary thrombi, swelling of endothelial cells, and mesangiolysis, which can lead to lumen stenosis or even occlusion. Ischemic shrinkage of the glomerulus occurs when the vascular lesions are severely involved. Arterial lesions are the most characteristic pathological manifestation of TMA. Arterioles may show arteriolar thrombi, fibrinoid necrosis, and subintimal myxoid edema in the acute phase. The typical “onion skin” pattern lesion is formed in the advanced stage (**Figure 1**). By EM, the swollen endothelial cells, widening subendothelial space, thickened glomerular basement membrane, and microthrombosis can be seen (5, 23, 25) (**Figure 2**).

### Immunosuppressive protocol and adjunctive therapies

Hydroxychloroquine (HCQ) is recommended for all LN patients without specific contraindications. Renin-angiotensin system inhibitors (RASi) can be used to control blood pressure and proteinuria. Under the standard induction period, glucocorticoids combined with cyclophosphamide (CYC) or mycophenolate mofetil (MMF), or calcineurin inhibitors (CNI) were used to treat patients with active Class III/IV ± V LN. Promising biological agents belimumab also can be chosen. Plasma exchange (PEX) was used in patients who showed clinical or pathological evidence of systemic TMA.

### Definitions

The starting point of follow-up was the time of kidney biopsy and was concluded when the last follow-up or end-point event occurred, which was defined as ESRD, regular dialysis, and death. Complete remission (CR) was defined as proteinuria < 0.5g/day and stable renal function, the latter indicated by a serum creatinine level not higher than 115% of baseline value. Partial remission (PR) was defined as decreasing proteinuria > 50% of the baseline value and < 3.5g/day and stable renal function. Those who did not meet CR or PR criteria were defined as treatment failure (26).

### Detection of renal MBL, MASP1/3, C4d, CFB, and CFD by immunofluorescence

Six patients classified as IV ± V LN with or without TMA were chosen for immunofluorescence and immunohistochemistry. Frozen kidney tissue sections were incubated with rabbit anti-human MBL polyclonal antibodies (1:20 dilution, Abcam, Cambridge, UK), rabbit anti-human MASP1/3 polyclonal antibodies (1:20 dilution; Proteintech, Wuhan, China), rabbit anti-human C4d polyclonal antibodies (1:300 dilution; Quidel, America), rabbit anti-human CFB polyclonal antibodies (1:20 dilution, Abcam, Cambridge, UK), and rabbit anti-human CFD polyclonal antibodies (1:20 dilution; Proteintech, Wuhan, China) for 2 hours at 37°C in a moist chamber. After washing these sections, FITC-conjugated affinipure secondary antibodies (1:30 dilution; Proteintech, Wuhan, China) were incubated for 50 minutes at 37°C. Ultimately, these slides were examined by the fluorescence microscope (Nikon, Tokyo, Japan). Semi-quantitative scoring of the results was performed by the professional pathologist under the same fluorescence photography conditions. 0 refers to no positive staining. 1, 2, and 3 refers to weak, moderate, and intense glomerular staining, respectively.

### Co-express of renal C5b-9 and CD34 detected by double-staining immunofluorescence

Add rabbit anti-human C5b-9 polyclonal antibodies (1:500 dilution; Abcam, Cambridge, UK) and mouse anti-human CD34 monoclonal antibodies (1:50 dilution; Proteintech, Wuhan, China) to frozen kidney tissue sections and incubated at 37°C for 2 hours. Then FITC/Cy3-conjugated affinipure secondary antibodies (1:30 dilution; Proteintech, Wuhan, China) were incubated at 37°C for 50 minutes. These sections were viewed eventually by a confocal laser scanning microscope and scored semi-quantitatively.

### Detection of VWF by immunohistochemistry

The minimal change disease (MCD) patients confirmed by renal biopsy were used as negative controls. Paraffin-embedded kidney biopsies were cut in 3 micrometer thick sections placed on slides, heat, deparaffinized by xylene, hydrated by gradient descent alcohol, recovered by microwave heating in citrate buffer (pH 6.0), and incubated in 0.3% hydrogen peroxide phosphate-buffered saline to inactivate endogenous peroxidase. Next, sections were stained with rabbit polyclonal antibodies for von Willebrand factor (VWF) (1:1200 dilution, Abcam, Cambridge, UK) overnight at 4°C in a moist chamber. Biotinylated secondary antibodies (1:100 dilution; Zhongshan Golden Bridge, Beijing, China) were incubated for 20 min at 37°C and an avidin-biotin-peroxidase complex (1:20 dilution; Zhongshan Golden Bridge, Beijing, China) was applied. Finally, diaminobenzidine was used as chromogen and hematoxylin as counterstaining. The staining for VWF was observed and assessed semi-quantitatively.

### Statistical analysis

Continuous variables were expressed as mean (standard deviation, SD) or median (range). Categorical variables were expressed as frequency (percentage). Normally distributed data were analyzed by Student’s t-test, while non-normal variables were compared by the Mann-Whitney U test appropriate. Categorical variables were compared using the x^2^ test or Fisher’s exact test when appropriate. Renal survival rates were estimated by the Kaplan-Meier method. Risk factors for renal outcomes were analyzed by multivariate Cox-regression analysis. All statistical analyses were performed by SPSS 25.0 (IBM Corporation, NY, USA) and Prism 8.0. P-values < 0.05 (two-tailed) were considered statistically significant.

## Results

### Patients’ general clinical characteristics and laboratory markers

104 patients with kidney biopsy showing LN and TMA were re-examined by two pathologists. 8 cases were excluded because the absence of clinical data. 17 cases were excluded because no evidence of TMA was observed under EM. Eventually, 79 LN patients showing renal TMA and 79 matched non-TMA controls were included in the analysis. The clinical characteristics of LN with or without TMA at the time of biopsy are listed in **Table 1**.

**Table 1:** Clinical characteristics of lupus nephritis patients with or without renal thrombotic microangiopathy.

Clinical characteristics	LN-TMA (n = 79)	LN (n = 79)	P-value
Patients demographics			
Sex (M/F)	11/68	11/68	1.0
Age (year)	29.37 ± 13.01	28.72 ± 10.86	0.735
Duration of SLE (months)	2 (1-192)	2 (1-132)	0.108
Duration of LN (months)	1 (1-192)	1 (1-84)	0.456
Duration of follow-up (months)	15.5 (1-84)	37 (1-45)	<0.001
Clinical parameters			
Hypertension (%)	68 (86%)	49 (62%)	0.001
Systolic arterial pressure, mmHg	143.95 ± 21.64	130.25 ± 18.49	<0.001
Diastolic arterial pressure, mmHg	93.10 ± 18.03	84.58 ± 14.17	0.001
Mean arterial pressure, mmHg	110.05 ± 18.17	99.81 ± 14.92	<0.001
Nephrotic syndrome (%)	46 (58%)	43 (54%)	0.630
Fever (%)	25 (32%)	15 (20%)	0.067
Mucosal ulcers (%)	4 (5%)	3 (4%)	0.699
Rash (%)	22 (28%)	25 (32%)	0.602
Hematuria (%)	65 (83%)	60 (76%)	0.328
Arthritis (%)	9 (11%)	10 (13%)	0.807
Serositis (%)	60 (76%)	41 (52%)	0.002
Neurological symptoms (%)	8 (10%)	2 (3%)	0.050
Acute heart failure (%)	20 (25%)	7 (9%)	0.006
Acute kidney injury (%)	24 (30%)	11 (14%)	0.013
Acute hemodialysis (%)	28 (35%)	4 (5%)	<0.001

Mean arterial pressure: diastolic arterial pressure + 1/3 pulse pressure; Nephrotic syndrome: proteinuria > 3.5g/day and albumin < 30g/l; Fever: > 38°C, exclude infectious cause; Mucosal ulcers: oral or nasal ulcerations; Rash: inflammatory type rash; Hematuria: > 5 red blood cells/high power field, exclude stone, infection or other cause; Arthritis: ≥ 2 joins with pain and signs of inflammation; Neurological symptoms: headache, cognitive disorder or seizure; Acute kidney injury: increase in SCr by ≥ 0.3mg/dl (≥ 26.5μmol/l) within 48 hours; or increase in SCr to ≥ 1.5 times baseline, which is known or presumed to have occurred with the prior 7 days; or urine volume < 0.5ml/kg/h for 6 hours.

As is shown in **Table 1**, of our 79 patients with renal TMA, females were accounted for 68 while males for 11, and aged 29.37 ± 13.01 years at the time of biopsy. Their SLE duration was 2 (range, 1~192) months and LN duration was 2 (range, 1~132) months. TMA patients presented a higher prevalence rate for hypertension, compared with non-TMA groups (86% vs. 62%, p = 0.001). In addition, patients with renal TMA showed higher systolic arterial pressure and diastolic arterial pressure (143.95 ± 21.64 mmHg and 93.10 ± 18.03 mmHg respectively) compared with non-TMA controls (130.25 ± 18.49 mmHg and 84.58 ± 14.17 mmHg respectively) (p < 0.001 and = 0.001 respectively). The incidence of serositis (76%), acute kidney injury (30%), and acute heart failure (25%) are higher in the cases with TMA (p = 0.002, 0.013, and 0.006 respectively). There are 28 (35%) lupus nephritis with TMA cases requiring acute hemodialysis compared to 4 (5%) non-TMA patients (p < 0.001). Neuropsychiatric SLE in TMA and non-TMA patients were 8 cases and 2 cases respectively (10% vs. 3%, p = 0.050). However, there was no difference in the prevalence of nephrotic syndrome, fever, mucosal ulcers, rash, hematuria, and arthritis between the two groups (p > 0.05, for all).

The comparison of baseline serological markers was also summarized in **Table 2**. SLE often affects the hematological system when it is active. Therefore, TMA cases that had higher SLEDAI scores (17.51 ± 4.75, compared with 15.08 ± 4.44 in non-TMA controls) had more serious anemia (p < 0.001) and lower platelets (p = 0.006). And microangiopathic hemolytic anemia (MAHA) was observed in 12 (15%) LN-TMA patients. In addition, more than half of TMA cases (59%) had a renal failure at the time of biopsy, while only 19% of LN patients had elevated serum creatinine. No statistical significance was observed in these serological indexes between LN patients with or without TMA, including proteinuria, albumin, C3 or C4 level, ESR, CRP, ANA antibody profiles, and antiphospholipid antibody (APLA). (p > 0.05, for all).

**Table 2:** Laboratory parameters of lupus nephritis patients with or without renal thrombotic microangiopathy.

Laboratory markers	LN-TMA (n = 79)	LN (n = 79)	P-value
Leukocytes (10^9^/l)	5.5 (1.5-13.91)	4.2 (1.0-17.9)	0.033
Hemoglobin (g/l)	84.54 ± 22.30	99.24 ± 19.86	<0.001
Platelets (10^9^/l)	115 (17-291)	144 (24-506)	0.006
Pancytopenia (%)	20 (25%)	13 (16%)	0.171
Thrombocytopenia (%)	34 (43%)	19 (24%)	0.011
Proteinuria (g/d)	4.64 (0.52-16.33)	3.82 (0.34-15.8)	0.194
Albumin (g/l)	25.72 ± 5.46	25.29 ± 6.84	0.663
Serum creatinine (μmol/l)	146 (53-1227)	72 (36-636)	<0.001
C3 level (g/l)	0.54 ± 0.27	0.52 ± 0.26	0.628
C4 level (g/l)	0.12 ± 0.09	0.10 ± 0.08	0.087
LDH^§^	378 (189-1364)	265 (127-1107)	0.002
MAHA (%)	12 (15%)	0 (0%)	<0.001
ANA seropositive (%)	77 (100%)	76 (99%)	1.0
Anti-dsDNA seropositivity (%)	55 (70%)	64 (82%)	0.097
Anti-dsDNA (IU/ml)	425.8 (10.0-1100.2)	592.36 (10.0-1140.7)	0.901
Anti-Smith seropositivity (%)	24 (31%)	28 (36%)	0.498
Anti-SSA-52 seropositivity (%)	40 (52%)	37 (48%)	0.633
Anti-SSA-60 seropositivity (%)	43 (56%)	37 (48%)	0.340
Anti-SSB seropositivity (%)	17 (22%)	11 (14%)	0.211
Anti-RNP seropositivity (%)	18 (26%)	27 (36%)	0.113
APLA seropositivity (%)^¶^ Total	3 (8%)	4 (15%)	0.631
Cholesterol (mmol/l)	5.75 ± 1.74	5.41 ± 2.15	0.284
Triglyceride (mmol/l)	2.84 ± 1.52	2.88 ± 1.80	0.881
ESR (mm/h)	39 (3.5-148)	39 (4.1-136)	0.519
CRP (mg/l)	2.99 (0-92.23)	2.95 (0-47.22)	0.326
SLEDAI score	17.51 ± 4.75	15.08 ± 4.44	0.001

Pancytopenia: Leukocytes, hemoglobin, platelets are lower than normal in peripheral blood; Thrombocytopenia: platelet count < 100 × 10^9^/l; LDH, lactic dehydrogenase; MAHA, microangiopathic hemolytic anemia; including low hemoglobin, schistocyte, elevated lactate dehydrogenase and indirect bilirubin, decreasing haptoglobin; APLA, antiphospholipid antibody includes anti-cardiolipin, anti-beta-2-glycoprotein I, and lupus anticoagulant. APLA seropositivity, > 40 MPL/GPL units; ESR, erythrocyte sedimentation rate; CRP, C-reactive protein; SLEDAI, Systemic Lupus Erythematosus Disease Activity Index 2000; §: 44 and 40 patients were tested LDH in LN with or without TMA, respectively; ¶: 38 and 27 cases were tested APLA in LN with or without TMA, respectively.

### Renal histological features

**Figure 3:**
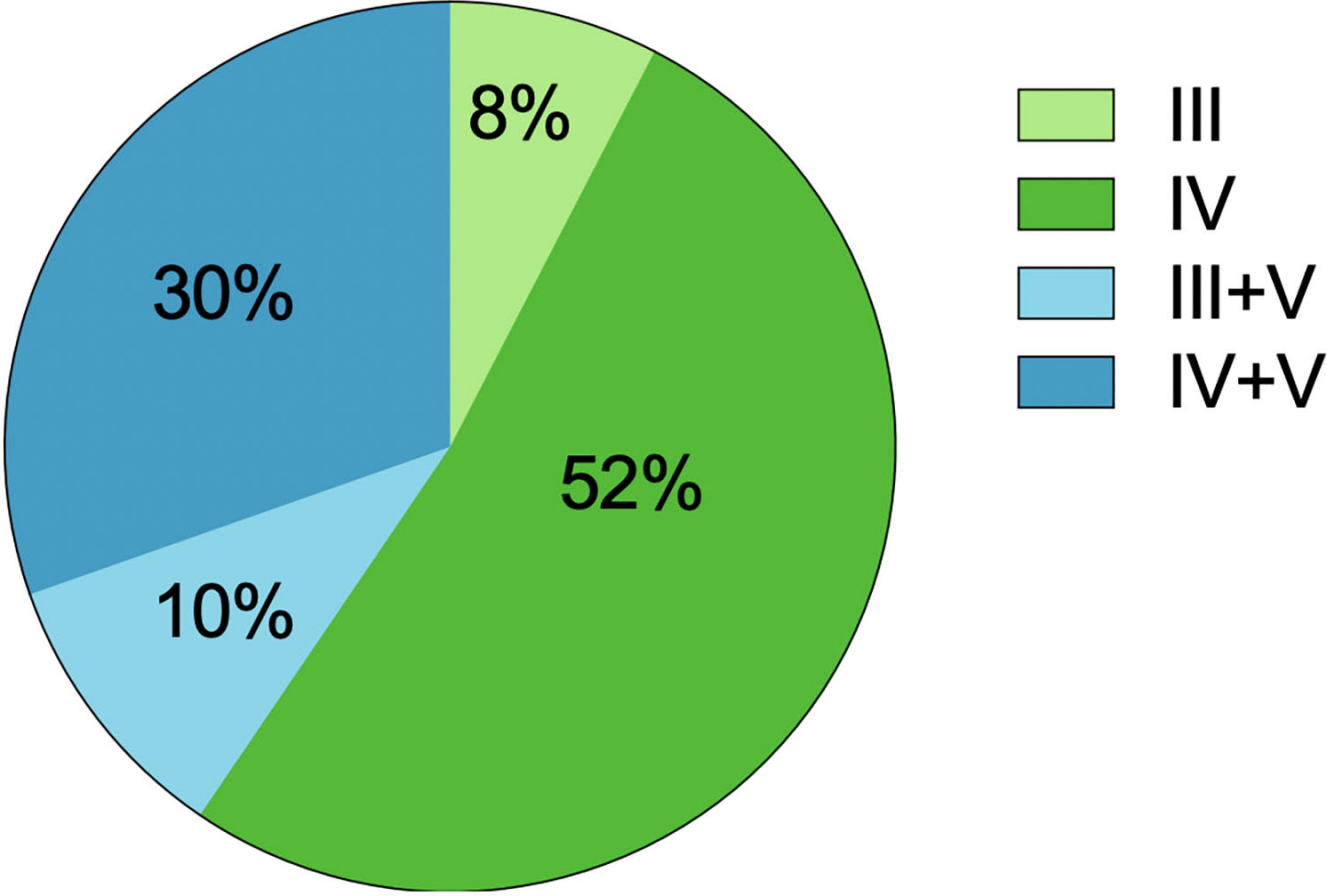
The distribution of lupus nephritis classes with TMA.

The renal histological features are detailed in **Table 3**. In our LN-TMA group, classes III, IV, III+V, and IV+V accounted for 8%, 52%, 10%, and 30% of cases; respectively (**Figure 3**). The activity index between the two groups was not statistically significant. However, the median scores of chronicity index were higher in the renal TMA group compared with the non-TMA group (p = 0.002). Among the CI-related indicators, the LN-TMA group had a higher proportion of fibrous crescents, tubular atrophy, and interstitial fibrosis (p = 0.001, 0.007, and < 0.001 respectively). The positive staining of the IgG, IgM, IgA, C3, C1q using immunofluorescence is denominated as “full house” pattern, which is common in LN and TMA.

**Table 3:** Renal histological features in lupus nephritis patients with or without thrombotic microangiopathy.

Renal histological features	LN-TMA (n = 79)	LN (n = 79)	P-value
Activity index	9.06 ± 3.61	8.10 ± 3.79	0.104
Chronicity index	1 (0-9)	0 (0-7)	0.002
Activity index			
Endocapillary proliferation (%)	79 (100%)	77 (97%)	0.156
Leucocyte infiltration (%)	65 (82%)	62 (78%)	0.549
Karyorrhexis (%)	19 (24%)	27 (34%)	0.163
Cellular crescents (%)	47 (59%)	39 (49%)	0.203
Microthrombus (%)	52 (66%)	41 (52%)	0.076
wire-loops (%)	50 (63%)	50 (63%)	1.0
Interstitial inflammation (%)	56 (71%)	53 (67%)	0.607
Chronicity index			
Glomerulosclerosis (%)	24 (30%)	30 (38%)	0.314
Fibrous crescents (%)	25 (32%)	8 (10%)	0.001
Tubular atrophy (%)	24 (30%)	10 (13%)	0.007
Interstitial fibrosis (%)	34 (43%)	10 (13%)	<0.001
Immunofluorescence			
IgG positive (%)	65 (82%)	69 (87%)	0.377
IgM positive (%)	49 (67%)	61 (81%)	0.049
IgM positive (%)	48 (61%)	64 (81%)	0.005
C3 positive (%)	65 (82%)	77 (97%)	0.002
C1q positive (%)	65 (82%)	71 (90%)	0.169

### Treatment protocols

All patients received oral prednisone. High-dose intravenous methylprednisolone pulse therapy was administered to 41 patients with TMA, compared with 26 cases without TMA (52% vs. 33%, p = 0.016, **Table 4**). Among 79 LN with TMA patients, 14 received PEX, much higher than the LN group (18% vs. 1%, p < 0.001). More TMA individuals chose CYC as induction therapy (27% vs. 13%, p = 0.028). However, there was no significant difference in the application of MMF and CNI between the two groups in the initial induction program.

**Table 4:** Treatment of lupus nephritis patients with or without renal thrombotic microangiopathy.

Treatment	LN-TMA (n = 79)	LN (n = 79)	P-value
Oral steroid (%)	79 (100%)	79 (100%)	1.0
Steroid pulse therapy (%)	41 (52%)	26 (33%)	0.016
RASi (%)	39 (49%)	47 (59%)	0.201
HCQ (%)	71 (90%)	58 (73%)	0.013
Steroids+CYC (%)	21 (27%)	10 (13%)	0.028
Steroids+MMF (%)	26 (33%)	26 (33%)	1.0
Steroids+MMF+CNI (%)	12 (15%)	9 (11%)	0.482
PEX (%)^‡^	14 (18%)	1 (1%)	<0.001

RASi, renin-angiotensin system inhibitors; HCQ, hydroxychloroquine; CYC, cyclophosphamide; MMF, mycophenolate mofetil; CNI, calcineurin inhibitors; PEX, plasma exchange. ‡: One patient in the control group underwent plasma exchange due to acute renal failure and hematologic system involvement. He eventually died of septic shock.

### Clinical outcomes

After the median follow-up of 15.5 (range, 1~84) months and 37 (range, 1~45) months in LN with or without TMA respectively, LN-TMA patients achieved inferior complete remission (CR) rate compared with non-TMA controls (22% vs. 63%, and 38% vs. 68%, at 6 and 12 months respectively, p < 0.001 for both, **Table 5**). Partial remission (PR) at 6 months of the TMA group was significantly lower than that of non-TMA controls (33% vs. 15% respectively, p = 0.008). However, the difference at 12 months did not reach statistical significance (20% vs. 15% respectively, p = 0.41). The patients in the LN-TMA group had significantly lower 2-year renal survival (73% vs. 90%, p = 0.008) and 3-year renal survival (68% vs. 89%, p = 0.002) rates than those in the non-TMA group. Nine individuals in the TMA groups developed the end-stage renal disease during the first month of treatment and seven died. While in the control group, one person needed regular dialysis and one died within the first month.

**Table 5:** Clinical outcomes of lupus nephritis patients with or without renal thrombotic microangiopathy.

Clinical outcomes	LN-TMA (n = 79)	LN (n = 79)	P-value
CR after 6 months	17 (22%)	50 (63%)	<0.001
PR after 6 months	26 (33%)	12 (15%)	0.008
CR after 12 months	28 (35%)	54 (68%)	<0.001
PR after 12 months	15 (29%)	12 (15%)	0.526
2-year renal survival	58 (73%)	71 (90%)	0.008
3-year renal survival	54 (68%)	70 (89%)	0.002

Annotations: Seven patients with renal TMA died within the first month, two because of acute heart failure, four due to pneumonia and one on account of septic shock. One case without renal TMA died due to sepsis shock.

### The risk factors

We used a COX proportional hazards model to analyze the risk factors which affect the prognosis of LN. Univariate COX regression analysis identified that TMA is a risk factor for renal survival in LN patients (HR 4.807, 95% CI [2.052, 11.263], p < 0.001). Hypertension (HR 12.044, 95% CI [1.643, 88.312], p = 0.014) is usually accompanied by a dismal prognosis, as did serositis (HR 2.634, 95% CI [1.083, 6.409], p = 0.033), acute kidney injury (HR 2.371, 95% CI [1.16, 4.849], p = 0.018), acute hemodialysis (HR 22.100, 95% CI [9.292, 52.560], p < 0.001), acute heart failure (HR 5.052, 95% CI [2.513, 10.154], p < 0.001), thrombocytopenia (HR 2.590, 95% CI [1.303, 5.148], p = 0.007) and interstitial fibrosis (HR 4.099, 95% CI [2.040, 8.238], p < 0.001) in LN. However, using RASi (HR 0.229, 95% CI [0.101, 0.519], p < 0.001) could improve renal survival rate. After adjusting for the influencing factors in the **Table 6**, multivariate Cox analysis found that acute hemodialysis (HR 7.089, 95% CI [1.130, 44.454], p = 0.037), acute heart failure (HR 3.605, 95% CI [1.186, 10.956], p = 0.024), and serum creatinine (HR 1.003, 95% CI [1.001, 1.006], p = 0.015) remained independent risk factors for renal outcome.

**Table 6:** Risk factors for renal survival determined by univariate/multivariate COX proportional hazard analysis in lupus nephritis.

Parameters	Univariate		Multivariate	
	HR (95% CI)	P-value	HR (95% CI)	P-value
TMA	4.807 (2.052, 11.263)	<0.001		
Hypertension	12.044 (1.643, 88.312)	0.014		
Systolic arterial pressure	1.021 (1.006, 1.037)	0.008		
Diastolic arterial pressure	1.032 (1.011, 1.053)	0.003		
Duration of SLE	1.008 (1.001, 1.015)	0.024		
Serositis	2.634 (1.083, 6.409)	0.033		
Acute kidney injury	2.371 (1.16, 4.849)	0.018		
Acute hemodialysis	22.100 (9.292, 52.560)	<0.001	7.089 (1.130, 44.454)	0.037
Acute heart failure	5.052 (2.513, 10.154)	<0.001	3.605 (1.186, 10.956)	0.024
Hemoglobin	0.973 (0.96, 0.986)	<0.001		
Thrombocytopenia	2.590 (1.303, 5.148)	0.007		
Serum creatinine	1.004 (1.003, 1.005)	<0.001	1.003 (1.001, 1.006)	0.015
CRP	1.017 (1.001, 1.034)	0.037		
SLEDAI score	1.120 (1.043, 1.203)	0.002		
RASi	0.229 (0.101, 0.519)	<0.001		
AI	1.099 (1.000, 1.207)	0.049		
CI	1.369 (1.202, 1.560)	<0.001		
Interstitial fibrosis	4.099 (2.040, 8.238)	<0.001		

Moreover, we used a COX proportional hazards model to evaluate the risk factors which affect the prognosis of LN-TMA (**Table 7**). Univariate COX regression analysis showed that acute hemodialysis (HR 19.473, 95% CI [5.572, 65.921], p < 0.001), acute heart failure (HR 3.525, 95% CI [1.605, 7.745], p = 0.002), thrombocytopenia (HR 2.627, 95% CI [1.170, 5.902], p = 0.019), glomerulosclerosis (HR 2.305, 95% CI [1.049, 5.064], p = 0.038) are risk factors for renal survival in LN-TMA patients. After adjusting the influencing factors in **Table 7**, acute hemodialysis (HR 8.719, 95% CI [1.319, 57.611], p = 0.025) was also an independent factor for renal survival.

**Table 7:** Risk factors for renal survival determined by univariate/multivariate COX proportional hazard analysis in LN-TMA.

Parameters	Univariate		Multivariate	
	HR (95% CI)	P-value	HR (95% CI)	P-value
Acute hemodialysis	19.473 (5.572, 65.921)	<0.001	8.719 (1.319, 57.611)	0.025
Acute heart failure	3.525 (1.605, 7.745)	0.002		
Hemoglobin	0.979 (0.964, 0.994)	0.006		
Platelets	0.992 (0.985, 0.999)	0.029		
Thrombocytopenia	2.627 (1.170, 5.902)	0.019		
Serum creatinine	1.003 (1.002, 1.004)	<0.001		
SLEDAI score	1.117 (1.033, 1.209)	0.006		
RASi	0.274 (0.109, 0.690)	0.006		
CI	1.214 (1.047, 1.408)	0.010		
Glomerulosclerosis	2.305 (1.049, 5.064)	0.038		

### Survival analysis

**Figure 4:**
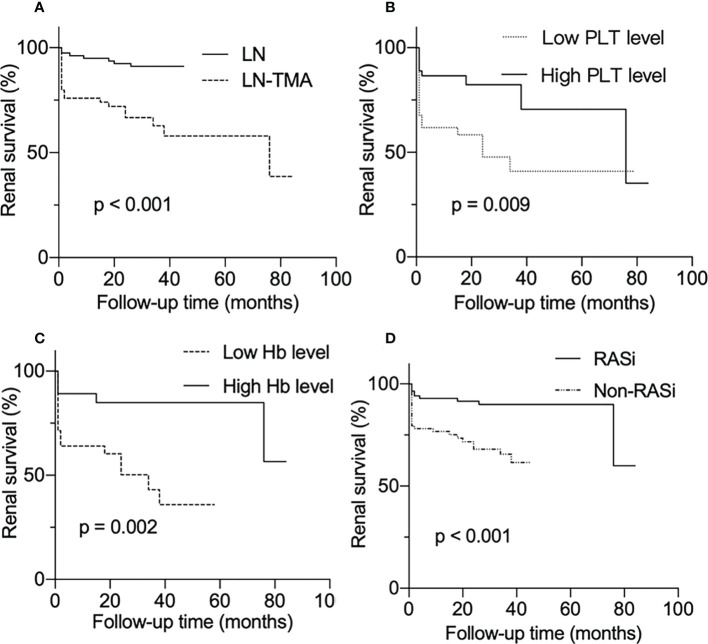
Renal survival rates of lupus nephritis patients. **(A)** With or without renal thrombotic microangiopathy among lupus nephritis patients. **(B)** Divided into low and high PLT level groups according to whether the platelet count is less than 100 × 10^9^/l among LN-TMA group. **(C)** TMA group was separated into low Hb and high Hb level according to whether Hb was less than 90g/l. **(D)** With or without RASi among LN patients.

As is shown in **Figure 4**, LN patients with renal TMA presented an inferior renal survival rate compared with non-TMA controls (p < 0.001, **Figure 4A**). TMA often involves the hematological system, manifesting as thrombocytopenia and anemia. The result showed that the prognosis of patients who had thrombocytopenia was worse (p = 0.009, **Figure 4B**). Similarly, TMA patients who showed Hb ≤ 90g/l had a poor prognosis (p = 0.002, **Figure 4C**). And using RASi is a protective factor for LN (p < 0.001, **Figure 4D**).

### The expression of MBL, MASP1/3, C4d, CFB, CFD, C5b-9, CD34, and VWF in kidney tissue

**Figure 5:**
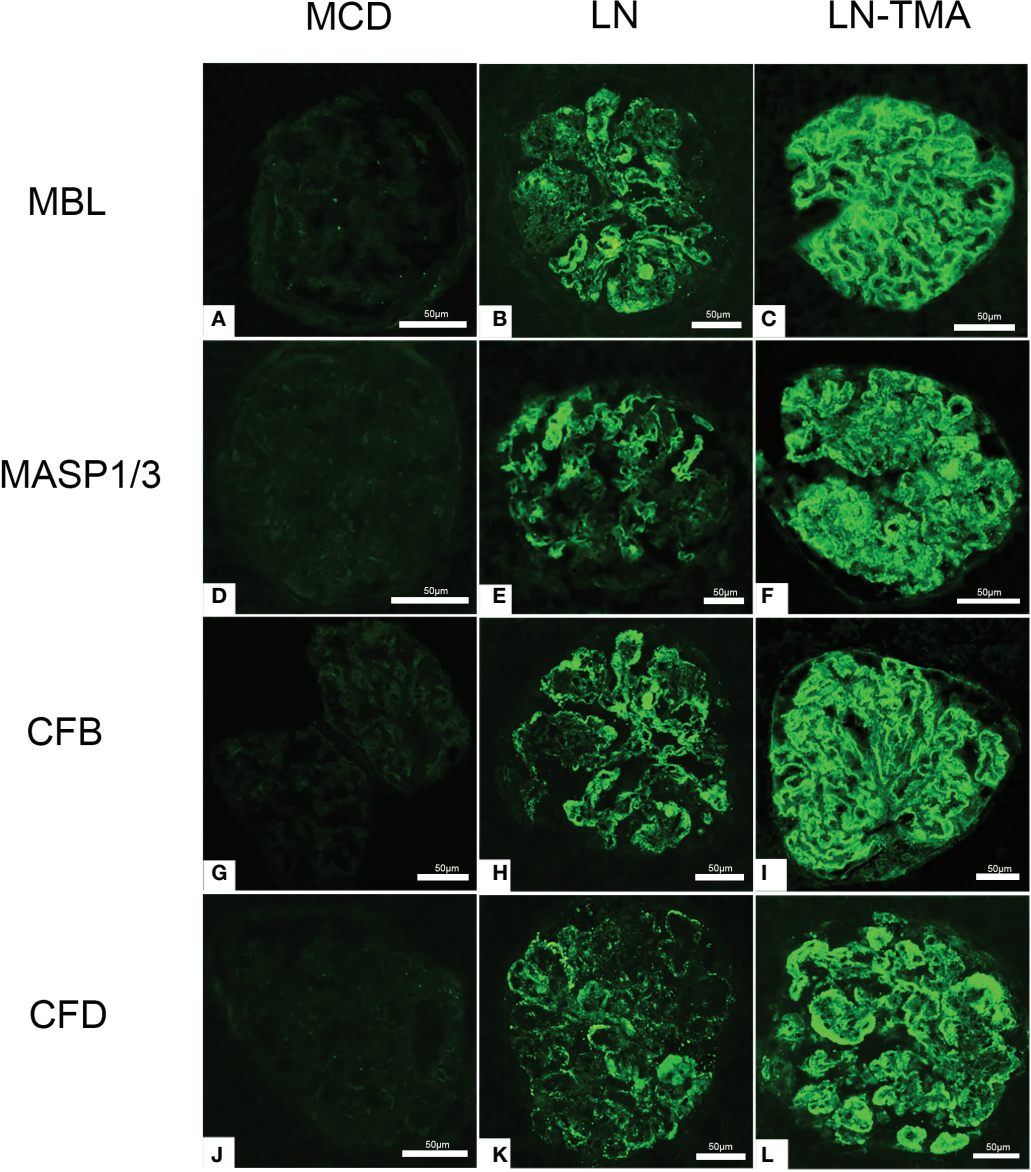
Immunofluorescence staining for MBL **(A-C)**, MASP1/3 **(D-F)**, CFB **(G-I)**, and CFD **(J-L)** among MCD, LN, and TMA groups. **(A, D, G, J)** Staining for MBL, MASP1/3, CFB, and CFD was negative in MCD renal tissues. **(B, E, H, K)** Moderate granular positive staining for MBL, MASP1/3, CFB, and CFD along the glomerular capillary and mesangial region in LN renal tissues. **(C, F, I, L)** Strong granular positive staining for MBL, MASP1/3, CFB, and CFD along the glomerular capillary and mesangial region in TMA renal tissues. Scale bars represent 50μm.

**Figure 6:**
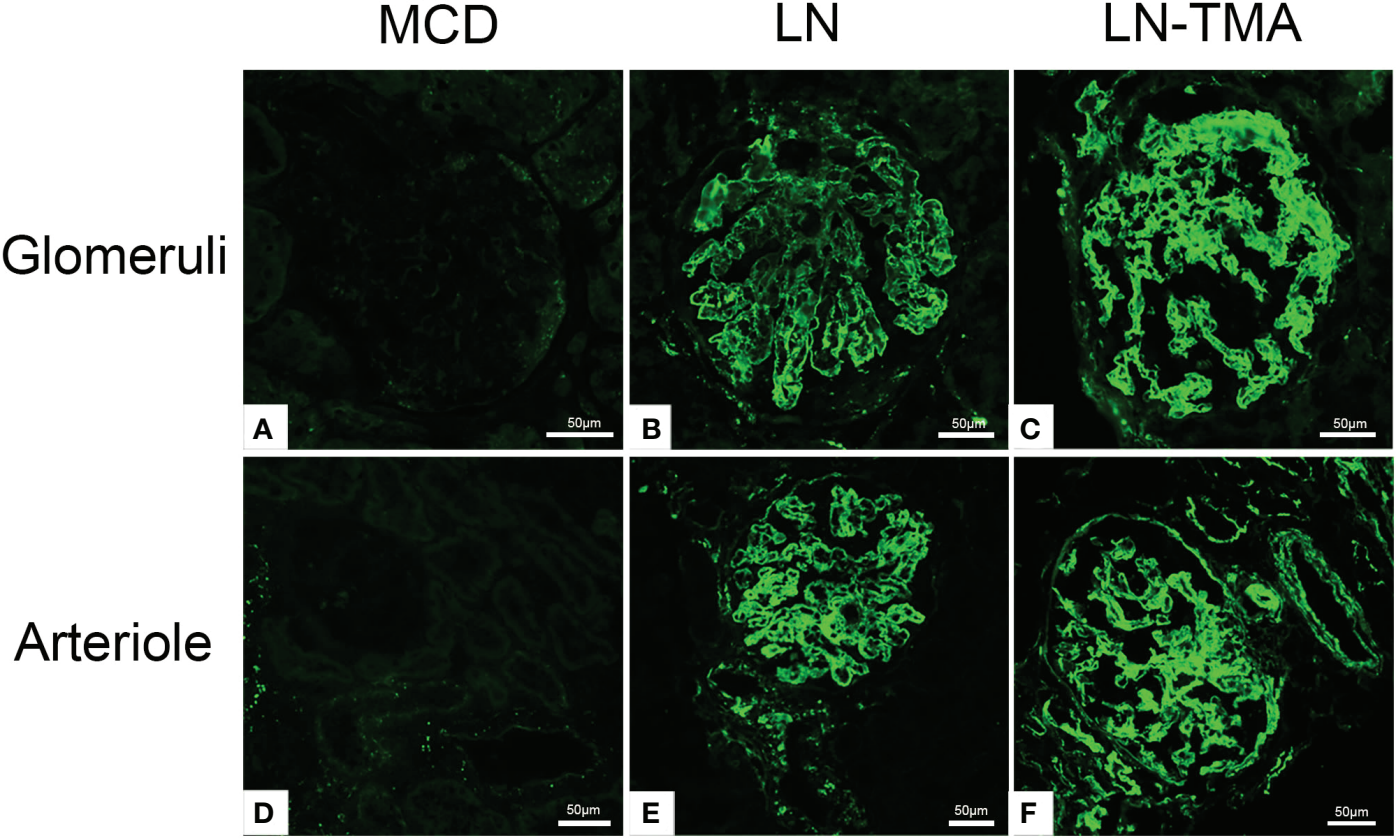
Frozen sections for C4d using immunofluorescence among MCD **(A, D)**, LN **(B, E)**, and TMA **(C, F)** patients. **(A, D)** Staining for C4d was negative in MCD renal tissues. **(B, E)** Moderate granular positive staining for C4d along the glomerular capillary and arteriole in LN renal tissues. **(C, F)** Strong granular positive staining for C4d along the glomerular capillary and arteriole in TMA renal tissues. Scale bars represent 50μm.

The LP activation elements MBL, MASP1/3, and the AP activation modules CFB, CFD deposited along the glomerulus and blood vessels (**Figure 5**). Compared with alone LN, the immunofluorescence staining intensity of patients with TMA was higher (**Supplementary Figure 1**), while the above complement components were not detected in the renal tissue of MCD patients. It is well known that activating the CP is the principal way to involve SLE progression (9). C4d is a fragment of C4 produced during complement activation and is considered as a symbol of classical and lectin pathway activation (27). Expression of C4d was strong in the LN group, especially in the renal arterials of TMA (**Figure 6**).

**Figure 7:**
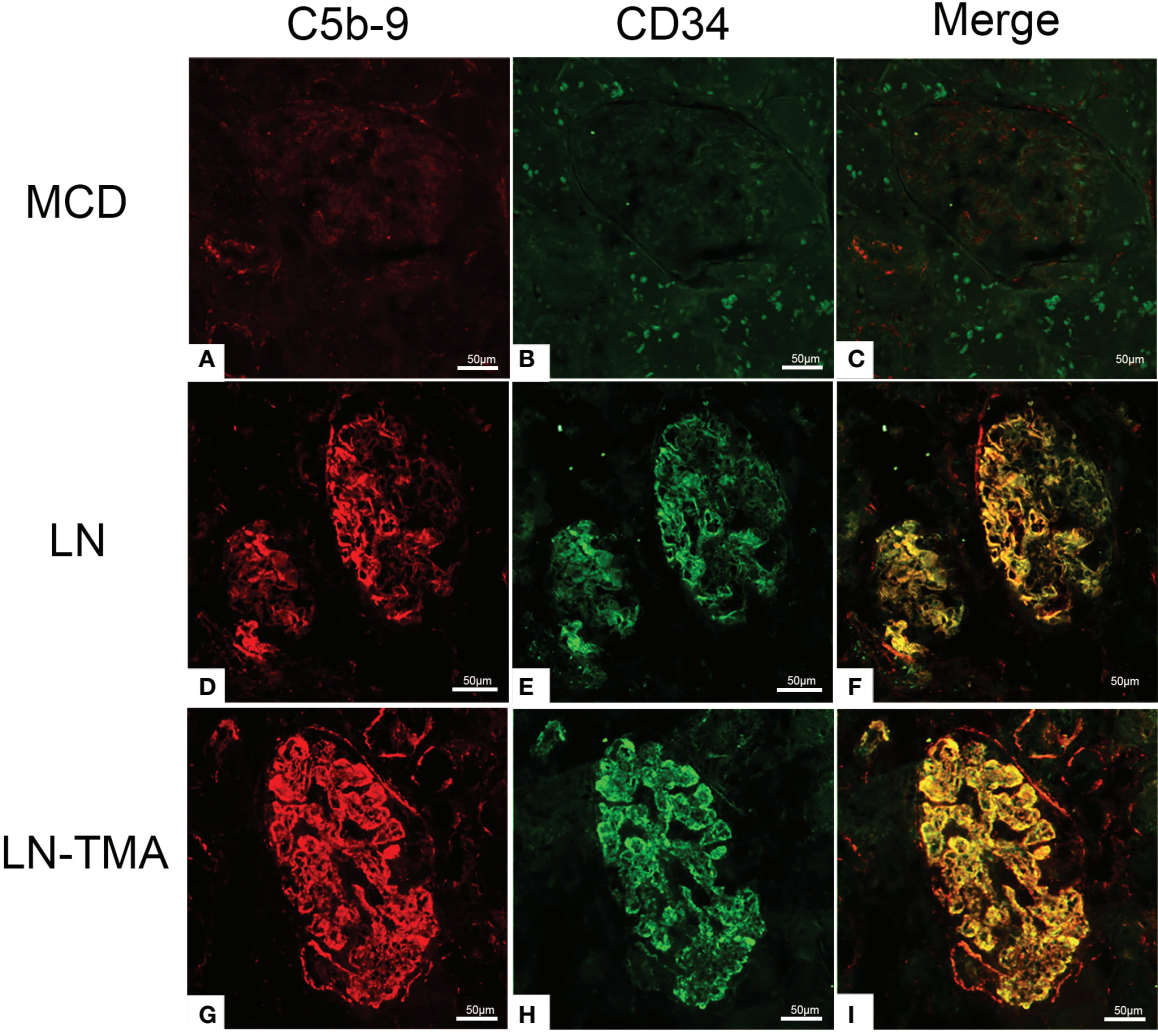
Frozen sections for C5b-9, CD34, and co-localization using immunofluorescence among MCD **(A-C)**, LN **(D-F)**, and TMA **(G-I)** patients. **(A-C)** Deposition of C5b-9 (red), CD34 (green), and co-localization (yellow) in MCD was negative. **(D-F)** Deposition of C5b-9 (red), CD34 (green), and co-localization (yellow) along the glomerular capillary and arteriole in LN was moderate staining. **(G-I)** Deposition of C5b-9 (red), CD34 (green), and co-localization (yellow) along the glomerular capillary and arteriole in TMA was strong staining. Scale bars represent 50μm.

**Figure 8:**
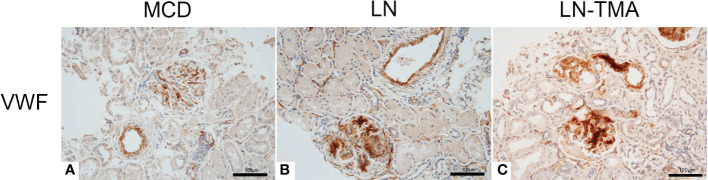
Paraffin sections for VWF **(A-C)** by immunohistochemistry among MCD, LN, and TMA patients. **(A-C)** The expression of VWF along the glomerular capillary and arteriole was strong, moderate, and weak in MCD **(A)**, LN **(B)**, and TMA **(C)** groups, respectively. Scale bars represent 100μm.

Using double-staining immunofluorescence, the co-localized expression of terminal complex of complement C5b-9 and endothelial marker CD34 in the glomerular and arterioles was observed in the renal tissue of LN patients with or without TMA (**Figure 7**). No positivity was observed for any of the studied indicators in MCD. The strong expression of VWF was detected in LN and TMA by immunohistochemistry, whereas weak staining was observed in MCD kidneys (**Figure 8**).

## Discussion

The incidence of TMA in LN kidney biopsies series range between 0.6% and 24.3% (7, 28, 29). In our retrospective cohort, we identified 79 renal TMA among 2130 biopsy-proven LN patients, occurring at a prevalence of 3.7%. In addition to ethnicity, genetics and environment, the significant variation may be also related to clinicians and pathologists deepening the understanding of TMA.

Our analysis results showed that patients with both renal TMA and LN had severer kidney injury features and poorer renal survival rates. Clinical characteristics including hypertension, serositis, acute kidney injury, acute hemodialysis, acute heart failure, anemia, thrombocytopenia, serum creatinine, and SLEDAI score; as well as renal histological indicators including chronicity index and interstitial fibrosis, are not only worse in TMA than those without TMA, but worthy variables to predict renal outcome in LN. Consistent with other previous studies, TMA was an independent risk factor for renal survival in LN patients (6, 7, 30–32).

TMA is characterized by thrombocytopenia, MAHA, and organ injury (33). The incidence of MAHA in TMA patients fluctuates from 8% to 60%, which is a valuable serum marker to assist in diagnosis and correlate with renal prognosis (34, 35). There were more TMA patients requiring acute hemodialysis at presentation due to elevated serum creatinine levels and various serious complications. Endocapillary lesions could be reversed by appropriate immunosuppression, which is related to renal function recovery (23). While interstitial fibrosis is the chronic damage that usually caused progressive renal insufficiency.

Renal vasculopathy in SLE are common, mainly due to the production of autoantibodies to form immune complex-induced vascular inflammation. There are many etiologies of renal TMA in lupus nephritis, including antiphospholipid syndrome, thrombotic thrombocytopenic purpura, malignant hypertension, pregnancy, systemic sclerosis overlap, drugs, and infections (26, 33). In addition, Christine et al. have revealed the importance of inherited and acquired disorders of complement in SLE and TMA (36). These gene mutations, including complement factor H, complement factor I, complement factor B, thrombomodulin, and MCP/CD46, contribute to occurring TMA (37).The clinical manifestations and treatment regimens caused by various causes are distinct (38, 39). KDIGO guidelines recommend PEX, glucocorticoids, rituximab, or caplacizumab in patients with ADAMTS13 (a disintegrin and metalloproteinase with a thrombospondin type I motif, member 13) activity < 10% (26). However, ADAMTS13 activity was not detected in this cohort. When antiphospholipid antibodies were positive and ADAMTS13 activity was normal, antiphospholipid syndrome nephropathy was considered and started anticoagulant therapy (26). We found that 6 cases in the TMA group were positive for antiphospholipid antibodies. Two additional patients presented with overlap syndrome, both overlapping with ANCA-associated vasculitis. In addition, no transplantation, malignancy, or drug-related TMAs were observed.

It is well known that the dysregulation of complement system is inextricably linked with SLE and TMA. Compared with LN alone, patients with TMA had superior serum Ba and C5b-9 levels (40). Besides, the plasma concentrations of MBL, MASP-1 and MASP-3 in SLE patients were higher than those in healthy controls (41). A study from Japan found that LN patients with MBL, L-ficolin, and properdin deposition had more urinary protein excretion. Meanwhile, patients with CFB and factor H deposition had the worse interstitial fibrosis (42). The MASP1 gene encodes MASP-1 and MASP-3, which are obtained by alternative splicing and translation of mRNA. Exons 1-8 and 10-11 encode the five N-terminal domains shared by MASP1 and MASP3. Exons 12 and 13-18 encode the linker and protease domains of MASP3 and MASP1, respectively (14). The role of MASP-1 in LP activation has been well understood, while Takahashi et al. found that sera from MASP1 gene knockout lupus-prone MRL/lpr mice (Masp1/3^-/-^ MRL/lpr mice) had little-to-no activation of both the LP and AP with zymogen forms of CFD (43). Furthermore, adding specific inhibitors for MASP-1 and MASP-3 to the plasma, CFD activation of the former was not affected, while the latter factor D existed as a precursor. Hence, MASP-3 was seen as a direct pro-CFD activator in resting blood (14). The influence of AP dysregulation on LN has been validated in murine models. Genetic deficiency of factor H in MRL/lpr mice presented marked albuminuria and azotemia. On the contrary, CFB deficient MRL/lpr mice had ameliorative clinical manifestations (20, 44, 45). The spectrum of C4d staining in glomerulus and arterioles was usual in both our and former studies (7, 32, 46, 47). Previous research found that C4d was a common denominator in TMA patients and arteriolar C4d deposition had prognostic value for the renal outcomes (32, 48). Vascular endothelial cell injury is a key link in the development of SLE and TMA (49). Co-localized expression of C5b-9 and CD34 confirmed the damage of complement activation to endothelial cells (12). The ultralarge VWF multimers secreted from damaged endothelial cells are released into the circulation and adhere to platelets to form microthrombi, leading to tissue ischemia, platelet consumption, and schistocytes (50, 51). Consequently, VWF deposition is strongly positive in TMA kidney tissues.

Despite treatment with glucocorticoids, immunosuppressive agents, and plasma exchange, the 3-year renal survival rate of TMA patients in this study was only 68%. Of the fourteen people underwent plasma exchange, six developed ESRD or died within the first month, suggesting that it is urgent to find novel biologic agents (52). 93% of TMA associated with LN patients had a favorable renal outcome under the premise of anti-C5 monoclonal antibody, eculizumab (53, 54). Moreover, Avacopan as an orally administered small-molecule C5a receptor inhibitor may be useful in LN patients (55). Narsoplimab, a monoclonal antibody against MASP-2, can inhibit LP activation and alleviate endothelial damage. Results from a clinical trial showed that it significantly improved remission and survival rate in patients with hematopoietic stem cell transplant-associated thrombotic microangiopathy (NCT047906), but its efficacy and safety in LN are still being tested (NCT02682407). LNP023, an invertible binding inhibitor of CFB, could alleviate pathological injury in the kidneys of MRL/lpr mice (56). In addition, CFB cleavage is abrogated in aHUS (atypical hemolytic uremic syndrome) patients’ serum when using danicopan, which is an oral CFD inhibitor (57).

There are some limitations in this study. Firstly, these enrolled individuals were mainly from Northern China, which is a single-center, retrospective research. Moreover, detecting complement-related autoantibody activity or gene mutations was needed. Furthermore, it is regrettable not to detect LP and AP-related complement components in serum and urine.

In conclusion, LN-associated TMA patients usually have more serious clinicopathological manifestations and inferior renal survival rates. LP and AP activation may play a crucial role in the pathogenesis of TMA in LN patients. This suggests that complement inhibition drugs can be widely used as novel therapeutic approaches in the future.

## Data availability statement

The original contributions presented in the study are included in the article/**Supplementary Material**. Further inquiries can be directed to the corresponding author.

## Ethics statement

The studies involving human participants were reviewed and approved by The first affiliated hospital of zhengzhou university. Written informed consent to participate in this study was provided by the participants’ legal guardian/next of kin.

## Author contributions

GX designed the study, BZ searched eligible literature, processing data and participated in the statistical analysis. BZ and GX drafted and revised the manuscript. All authors contributed to the article and approved the submitted version.
