# Supplementary figures and images for: Thrombotic microangiopathy mediates poor prognosis among lupus nephritis via complement lectin and alternative pathway activation
Source: Front Immunol. 2022 Dec 13;13:1081942. doi: 10.3389/fimmu.2022.1081942 (PMC9792970; doi:10.3389/fimmu.2022.1081942)

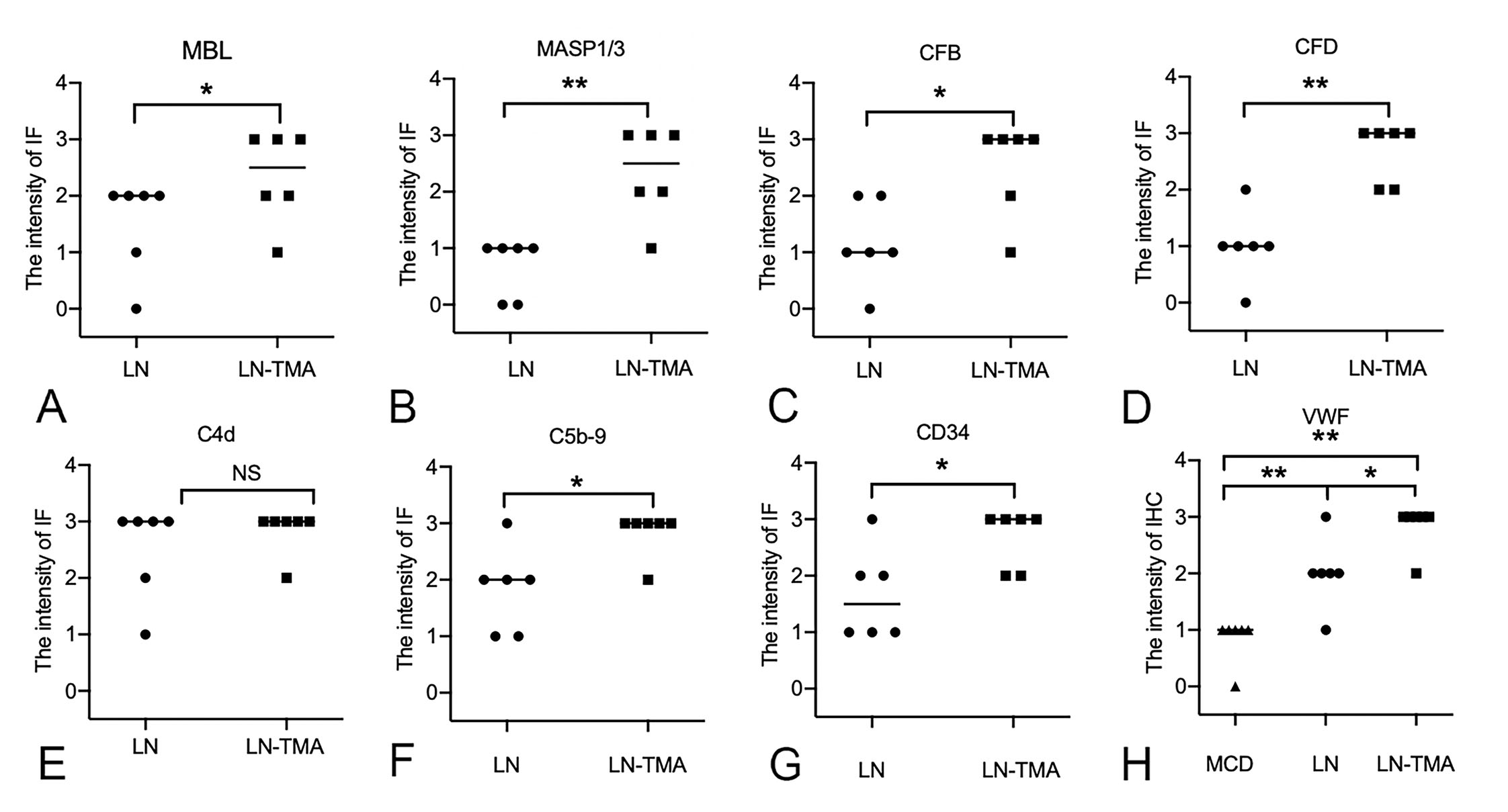

Supplement: Supplementary file 1 [file Image_1.jpeg]
